# Submillimeter Diameter Poly(Vinyl Alcohol) Vascular Graft Patency in Rabbit Model

**DOI:** 10.3389/fbioe.2016.00044

**Published:** 2016-06-08

**Authors:** Marie F. A. Cutiongco, Marek Kukumberg, Jonnathan L. Peneyra, Matthew S. Yeo, Jia Y. Yao, Abdul Jalil Rufaihah, Catherine Le Visage, Jackie Pei Ho, Evelyn K. F. Yim

**Affiliations:** ^1^Mechanobiology Institute, National University of Singapore, Singapore; ^2^Department of Biomedical Engineering, National University of Singapore, Singapore; ^3^Comparative Medicine, National University of Singapore, Singapore; ^4^Division of Plastic, Reconstructive and Aesthetic Surgery, Department of Surgery, National University Health System, Singapore; ^5^Plastic, Reconstructive and Aesthetic Surgery Section, Department of General Surgery, Tan Tock Seng Hospital, Singapore; ^6^Department of Surgery, Yong Loo Lin School of Medicine, National University of Singapore, Singapore; ^7^INSERM, U791, Center for OsteoArticular and Dental Tissue Engineering, Université de Nantes, Nantes, France; ^8^Department of Cardiovascular and Thoracic Surgery, National University Health System, Singapore; ^9^Department of Chemical Engineering, University of Waterloo, Waterloo, ON, Canada

**Keywords:** small diameter vascular graft, *in vivo* testing, compliance matching, off-the-shelf vascular graft, microvascular graft

## Abstract

Microvascular surgery is becoming a prevalent surgical practice. Replantation, hand reconstruction, orthopedic, and free tissue transfer procedures all rely on microvascular surgery for the repair of venous and arterial defects at the millimeter and submillimeter levels. Often, a vascular graft is required for the procedure as a means to bridge the gap between native arteries. While autologous vessels are desired for their bioactivity and non-thrombogenicity, the tedious harvest process, lack of availability, and caliber or mechanical mismatch contribute to graft failure. Thus, there is a need for an off-the-shelf artificial vascular graft that has low thrombogenic properties and mechanical properties matching those of submillimeter vessels. Poly(vinyl alcohol) hydrogel (PVA) has excellent prospects as a vascular graft due to its bioinertness, low thrombogenicity, high water content, and tunable mechanical properties. Here, we fabricated PVA grafts with submillimeter diameter and mechanical properties that closely approximated those of the rabbit femoral artery. *In vitro* platelet adhesion and microparticle release assay verified the low thrombogenicity of PVA. A stringent proof-of-concept *in vivo* test was performed by implanting PVA grafts in rabbit femoral artery with multilevel arterial occlusion. Laser Doppler measurements indicated the improved perfusion of the distal limb after implantation with PVA grafts. Moreover, ultrasound Doppler and angiography verified that the submillimeter diameter PVA vascular grafts remained patent for 2 weeks without the aid of anticoagulant or antithrombotics. Endothelial cells were observed in the luminal surface of one patent PVA graft. The advantageous non-thrombogenic and tunable mechanical properties of PVA that are retained even in the submillimeter diameter dimensions support the application of this biomaterial for vascular replacement in microvascular surgery.

## Introduction

Microvascular surgery contributes widely to various surgical procedures, such as replantation and free tissue transfer (Griffin and Thornton, 2005), free flap surgery (Shen et al., 1988), orthopedic surgeries (Doi et al., 1977; Judet et al., 1981), and digital reconstruction (Lanzetta, 1995). During these procedures, vascular grafts are required to anastomose blood vessels together, repair vascular defects, and restore blood flow. The gold standard of using autologous venous and arterial grafts, which is valued for their biocompatibility and non-thrombogenicity, is not without fault. Autologous grafts require tedious and time-consuming harvesting processes and may be limited supply due to comorbidities that affect the vasculature. Moreover, venous autologous grafts lack mechanical integrity while autologous grafts have a propensity for aneurysm formation (So, 1998).

Synthetic small diameter vascular grafts (with diameter <6 mm) made of expanded polytetrafluoroethylene (ePTFE) are widely available today as alternatives to autologous vascular grafts. However, ePTFE grafts are plagued by rapid thrombosis and lack of long-term patency and efficacy caused by mechanical mismatch with the native artery (Uchida et al., 1989; Sarkar et al., 2007). The problems of ePTFE grafts are further magnified in the submillimeter scale, where the low shear stress and flow, and high risk of blood stasis can lead to rapid graft thrombosis and failure (Barnes, 1980; Sarkar et al., 2006). ePTFE grafts with 1 mm diameter have all failed after implantation *in vivo*, showing rapid and extensive thrombosis within a few days of implantation (Lidman et al., 1980; Ganske et al., 1982; Harris and Seikaly, 2002). Thus, the development of a new type of off-the-shelf synthetic vascular graft with submillimeter diameter for microvascular surgery is needed.

Poly(vinyl alcohol) hydrogel (PVA) is widely used for various biomedical purposes, including the production of artificial meniscus (Kobayashi et al., 2005), hemodialysis membrane (Barzin and Madaeni, 2007), heart valve (Wan et al., 2002), artificial vitreous fluid (Lamponi et al., 2012; Feng et al., 2013), and cardiac patch with drug delivery capability (Fathi et al., 2013). PVA is an excellent candidate as a small diameter vascular graft due to its non-immunogenic, bio-inert, and mechanically tunable properties (Chaouat et al., 2008; Cutiongco et al., 2015a, 2016). Herewith, we sought to tune the mechanical properties of submillimeter diameter PVA grafts. Characterization of mechanical and thrombogenic properties of the submillimeter PVA grafts will be performed. Demonstration of efficacy of PVA graft in stringent *in vivo* conditions submillimeter diameter will reiterate the potential of this material as a new vascular graft material for microvascular surgical applications.

## Materials and Methods

### Fabricating PVA Vascular Grafts

Fabrication of grafts made from PVA hydrogel (PVA graft) was performed according to previous studies (Cutiongco et al., 2015b, 2016). Hypodermic needle [0.9 mm outer diameter (OD)] and carbon rod (1 mm OD) were used to fabricate PVA vascular grafts. Cylindrical molds were plasma cleaned (60 W, 8 mL/min O_2_ gas) for 1 min to improve adherence of PVA. The mold was then immediately immersed in solution of PVA, crosslinker STMP, and NaOH, removed, and dried for 15 min at room temperature. An additional five layers of PVA were added and allowed to dry at 25°C for 3 days or 4°C for 3 days. Afterward, scaffolds were washed several times in phosphate-buffered saline (PBS) and distilled deionized (DI) water before removal from the tubular mold. PVA tubes were gamma irradiated (25 kGy) while immersed in water to obtain sterile PVA vascular grafts.

### Mechanical Testing of Submillimeter PVA Grafts

#### Wall Thickness and Internal Diameter Measurement

Different sections of PVA vascular grafts (*n* = 5) hydrated in water were cut into ~1 mm cross-sections and imaged using optical microscope (Nikon Eclipse TS100). The images were used to measure wall thickness and ID using ImageJ (NIH).

#### Radial Compliance Test

At hydrostatic pressures of 80 and 120 mmHg, images of the distended PVA vascular grafts (*n* = 6) were taken using a stereomicroscope (Nikon SMZ745T). Compliance was calculated as the percent change in PVA vascular graft diameter from 80 to 120 mmHg (Chaouat et al., 2008).

#### Burst Pressure Test

Nitrogen gas was slowly released into closed-ended PVA vascular grafts (*n* = 3) until scaffold rupture. Pressure at failure was denoted as the burst pressure.

#### Suture Retention Test

A single throw of 6/0 Vicryl suture (Ethicon) was passed through one end of a PVA vascular graft at ~5 mm from the edge. The suture was attached to a receptacle, where water was added until rupture of the PVA graft. The weight of the receptacle and water was taken as suture retention strength (*n* = 4).

#### Uniaxial Test

PVA vascular grafts (1 cm length, *n* = 3) were tested using a uniaxial testing machine (INSTRON 3345) with a 10-N load cell, 0.1-N preload, and crosshead speed of 10 mm/min. Measurements of various grafts were taken until break. Linear portions of the graphs were used to determine Young’s modulus.

### Scanning Electron Microscopy of Submillimeter PVA Grafts

PVA vascular graft was air-dried overnight at ambient temperature, sputter coated with 10 nm platinum film (JEOL-JFC 1600 coater), and visualized using scanning electron microscopy (SEM) (JEOL-JSM 6010LV).

### Blood Compatibility of PVA

#### Incubation of PVA Tubes with Platelet Rich Plasma

*In vitro* blood compatibility assay was performed, as previously described (Cutiongco et al., 2015a). Blood samples were collected from healthy New Zealand White rabbits in polypropylene tubes primed with heparin (5 U/mL blood). Blood was centrifuged at 100 × *g* and 22°C for 12 min to collect platelet rich plasma (PRP). PVA vascular graft (1 mm ID, *n* = 3, internal surface area of 90 mm^2^), Silastic tubing (Dow Corning, *n* = 3, internal surface area of 60 mm^2^), and ePTFE tubing (Zeus, *n* = 3, internal surface area of 110 mm^2^) were weighed and washed in sterile 0.9% NaCl solution (BBraun). Glass beads coated with bovine Collagen I (Invitrogen; 7.15 μg/mg sample) were placed in a polypropylene tube and included as a positive control. PRP was then added to the lumen of each tube or to the beads at 0.75 μL/mg sample. Since the diameters of the tubes and beads used in this test were different, a constant mass of each sample was used. Tubes were sealed at both ends with sterile Luer lock combistoppers (BBraun). Subsequently, all samples were incubated for 1 h at 37°C with bidirectional *X*-axis rotation at 100 rpm. Resting samples of 100 μL PRP were also included. Tubular samples were kept for platelet morphology analysis using SEM (see Preparation of Samples for SEM Visualization), while PRP was then collected for subsequent flow cytometry analysis (see Flow Cytometric Analysis of Platelet Activation). Assay was done in triplicate for each sample.

#### Preparation of Samples for SEM Visualization

Tubular samples were washed in PBS and fixed using 2.5% glutaraldehyde at 4°C for 2 h. Thereafter, films were dehydrated using a series of increasing ethanol concentration. After complete drying, films were then coated and visualized under SEM, as described in Section “Scanning Electron Microscopy of Submillimeter PVA Grafts.”

#### Flow Cytometric Analysis of Platelet Activation

Aliquots of PRP (10 μL) were diluted with 200 μL of HEPES-Tyrodes buffer (HTB; 137 mM sodium chloride, 2.7 mM potassium chloride, 16 mM sodium bicarbonate, 5 mM magnesium chloride, 3.5 mM HEPES, 1% glucose, 2% bovine serum albumin, and pH 7.4) and incubated with antibodies against CD61/GPIIb/IIIa (Abbiotec). Platelets were then washed with HTB and centrifuged at 21,000 rpm and 4°C for 5 min. Platelets were then resuspended in 100 μL HTB and incubated with fluorescently conjugated secondary antibodies for 1 h. Samples were fixed with 2% paraformaldehyde in HTB. Platelets were analyzed within 24 h for scatter characteristics and expression of platelet markers using BD LSR Fortessa. Flow cytometry data were analyzed using FlowJo 4.0.

### PVA Vascular Graft Implantation in Rabbit Femoral Artery with Multilevel Arterial Occlusion

Animal study was done in accordance with approved guidelines of the Institutional Animal Care and Use Committee of the National University of Singapore. Male New Zealand White rabbits (3.5–4.0 kg, ~6 months old) were used in the study. Multilevel arterial occlusion in the left femoral artery was induced through embolization and ligation of the femoral artery before PVA graft anastomosis (Figure S1 in Supplementary Material).

After sedation and exposure of the left femoral artery, papaverine (1.43 mg/mL) was administered topically and intravascularly to facilitate intra-arterial cannulation. Embolic particles (150–250 μm diameter, Boston Scientific) resuspended in equal volume of 0.9% sodium chloride and 4% gelofusine were injected into the left femoral artery. In a pilot study, the minimum dose of embolic particles needed to induce occlusion of the lower saphenous artery was determined by titration (data not shown). The embolic particles were flushed into the femoral artery with 0.9% NaCl solution. The femoral artery was permanently ligated. A 4-mm length, encompassing the site of intra-arterial cannulation and found between the ligatures, was resected to induce macro-vascular occlusion, thereby completing the induction of the multilevel arterial occlusion.

Immediately afterward, PVA vascular grafts with 4-mm length and 0.9- or 1-mm ID were implanted into the left femoral artery (PVA group). The size of graft implanted was chosen to match the caliber of the femoral artery. PVA vascular grafts were anastomosed using simple interrupted sutures (Ethilon, 10-0) under operating stereomicroscope. During anastomosis, heparin (100 IU/kg) was administered intravenously. Ligation and reperfusion of left femoral artery did not exceed 3 h in all surgeries. Postanastomotic patency was confirmed by refill test and observation of pulsation in the postanastomotic vessel. All animals were treated with enrofloxacin (5 mg/kg) and buprenorphine (0.04 mg/kg) for 7 days postoperatively. The contralateral hindlimbs in each animal were included as an internal control (contralateral group, *n* = 7).

### Assessment of Hindlimb Perfusion Using Laser Doppler Flowmeter

The perfusion of the skin on the left dorsal foot was measured using laser Doppler flowmeter (Perimed PeriFlux System 5010 with 1 mm diameter fiber-optic probe). Laser Doppler flowmetry measurement of surface paw perfusion was performed immediately after surgery and at intervals afterward. Animals were sedated before measurement. To ensure consistency, laser Doppler probe was placed on the same location on the depilated dorsal foot, as marked by a small circular tattoo (AIMS animal tattoo). Measurements of surface perfusion were obtained from the stable readout of the laser Doppler flowmeter. All dorsal foot perfusion measurements were normalized to that of the contralateral group. For comparison, standard laser Doppler imaging (Perimed PeriFlux PIM 3) was performed and confirmed the validity of the readings obtained from the laser Doppler flowmeter (Figure S2 in Supplementary Material).

### Ultrasound Doppler

Examination of vascular graft patency during implantation was performed using ultrasound Doppler (GE Ultrasound Doppler Vivid S6, 8–13 MHz). Animals were lightly sedated and placed in prone position during the examination.

### Endpoint Angiography

Digital subtraction angiography (GE Innova 2100) was performed at ~15 days postimplantation of the PVA grafts. Contralateral limbs were included as the internal control. Contrast agent (GE Omnipaque, 350 mg/mL) and 0.9% NaCl solution at a 1:1 ratio were used for imaging. A midline incision was made on the sedated rabbit. The caudal abdominal aorta was cannulated near the iliac bifurcation for direct delivery of contrast agent through each iliac artery. Quantification of angiographic data was performed, as described previously (Silvestre et al., 2000; Iglarz et al., 2001).

### Histology

Heparin (100 IU/kg) was administered systemically before euthanization with sodium pentobarbital (150 mg/kg). Muscle from both the implanted and contralateral limbs were harvested and fixed with 4% paraformaldehyde for 48 h. Tissues were embedded in Paraplast (Leica). Sections 15–20 μm thick were stained with standard hematoxylin and eosin (H&E) and Masson’s trichrome stain. Assessment of collateral formation and graft endothelialization was performed through detection of CD31 and α-SMA using Novolink kit (Leica).

### Statistical Analysis

All data are presented as mean ± SD. Statistical analysis was performed using one-way ANOVA with Tukey’s *post hoc* test (GraphPad Prism v6.0). Differences were taken to be statistically significant at *p* < 0.05.

## Results

### Tuning Mechanical Properties of Submillimeter Diameter Vascular Grafts

The lack of clinically available submillimeter diameter vascular grafts necessitates the creation of a new vascular graft. Thus, PVA submillimeter diameter vascular grafts were fabricated. Matching size and compliance of a synthetic vascular graft to the native recipient vessel is critical to maintain graft patency (Uchida et al., 1989; Sarkar et al., 2006). To better approximate mechanical properties of the rabbit femoral artery, PVA vascular grafts with uniform ID of 0.9 and 1 mm were fabricated at two different cross-linking temperatures of 25 and 4°C (Table 1).

**Table 1:** **Mechanical properties of poly(vinyl alcohol) (PVA) grafts with submillimeter dimensions and reported values for rabbit femoral artery**.

Tube	Internal diameter (μm, *n* = 5)	Wall thickness (μm, *n* = 5)	Compliance (%, *n* = 6)	Burst pressure (mmHg, *n* = 3)	Suture retention (g, *n* = 4)	Young modulus (kPa, *n* = 3)	Elongation at break (%, *n* = 3)	Ultimate tensile strength (kPa, *n* = 3)
**Cross-linking at 25°C**								
1 mm diameter	1194 ± 29.62^a^ (hydrated)	271.2 ± 70.47	4.00 ± 3.41	725 ± 137^c^	102 ± 16.9	553.1 ± 2.695^b^,^c^	436.0 ± 25.36^c^	84.4 ± 13.4^b^,^c^
0.9 mm diameter	1093 ± 63.61 (hydrated)	287.6 ± 27.59	3.04 ± 2.56	868 ± 171^c^	76.6 ± 7.33^a^	388.7 ± 34.28^b^	465.8 ± 21.72^c^	53.7 ± 11.7^b^
**Cross-linking at 4°C**								
1 mm diameter	1185 ± 9.510^a^,^b^ (hydrated)	273.8 ± 50.71	2.87 ± 0.14	269 ± 134^c^	90.3 ± 13.7	256.9 ± 61.34^c^	161.6 ± 41.22^c^	57.1 ± 9.90^c^
0.9 mm diameter	996.6 ± 29.85^b^ (hydrated)	285.1 ± 47.74	3.29 ± 0.63	207 ± 104^c^	74.8 ± 5.90^a^	331.9 ± 90.54	130.7 ± 19.84^c^	44.0 ± 2.83
Rabbit femoral artery (Uchida et al., 1989)	938 ± 201	350–710	5.9 ± 0.5	2031–4225	200 ± 119	230	ND	998

*^a^Denotes statistical significance between PVA graft and rabbit femoral artery*.

*^b^Denotes statistical significance between 1 mm internal diameter (ID) PVA graft and 0.9 mm ID PVA graft cross-linked at the same temperature*.

*^c^Denotes statistical significance of PVA graft with similar ID cross-linked at different temperatures*.

The internal diameter (ID) and wall thickness between PVA grafts cross-linked at different temperatures but with similar ID showed minute differences. On the other hand, compliances of grafts cross-linked at 4°C were generally lower than those of tubes cross-linked at 25°C. Furthermore, burst pressure of grafts were significantly improved when grafts were cross-linked at 25°C compared to 4°C, showing at least a 2.7 times increase in magnitude. Both Young’s modulus and ultimate tensile strength of 1 mm ID PVA grafts were invariably improved when cross-linking temperature changed from 4 to 25°C. Similar to the observed improvement in burst pressure, the percent elongation at break was also drastically changed when cross-linking temperature was increased from 4 to 25°C.

**Figure 1:**
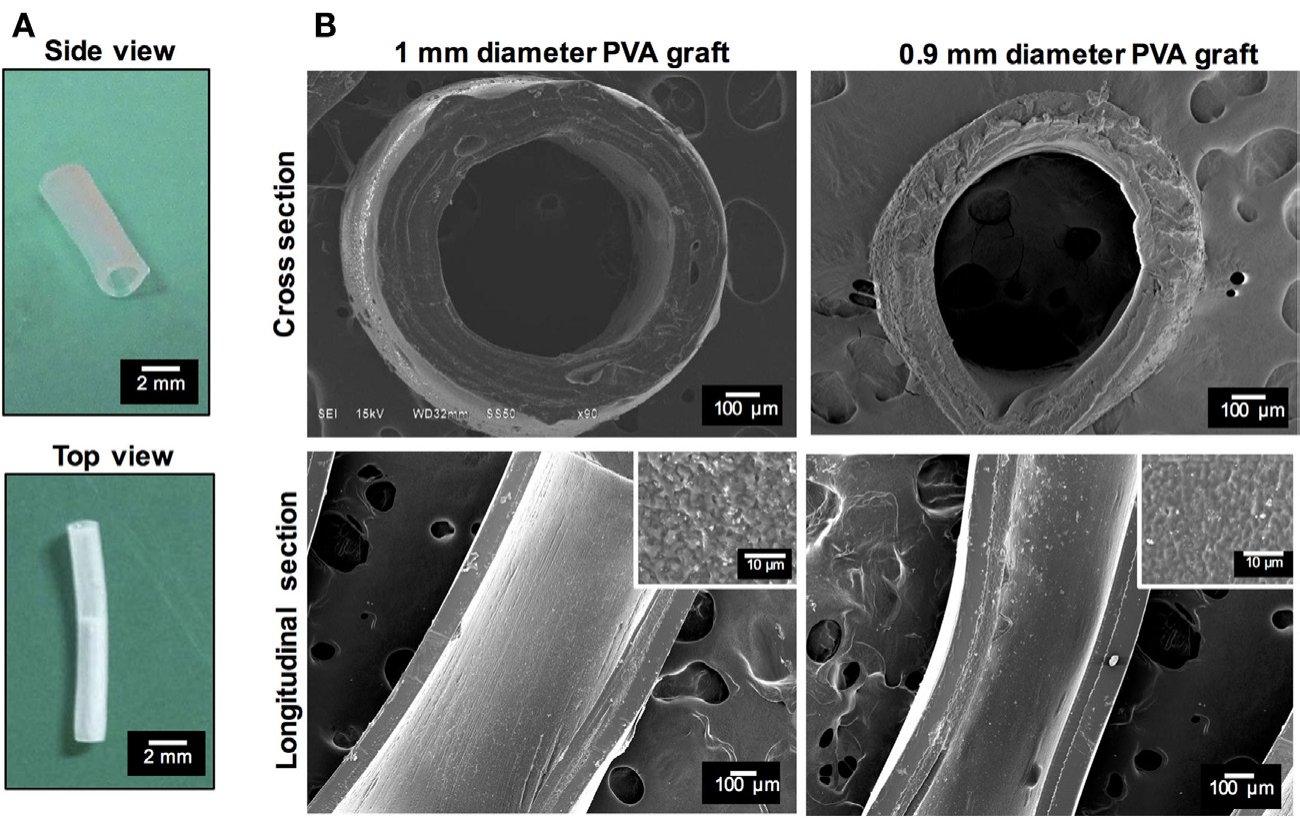
**Characterization of submillimeter diameter poly(vinyl alcohol) (PVA) vascular graft**. **(A)** Photomicrograph of PVA vascular graft with 1 mm internal diameter. **(B)** Scanning electron microscopy (SEM) images show cross section and longitudinal section of 1 and 0.9 mm ID PVA vascular grafts cross-linked at 25°C. The longitudinal section shows surface roughness on lumen.

Due to the compliance more closely approximating the rabbit femoral artery, together with better mechanical integrity as indicated by higher burst pressure and ultimate tensile strength, PVA grafts cross-linked at 25°C were chosen for implantation in the rabbit femoral artery. Under SEM, the PVA vascular grafts cross-linked at 25°C (afterward referred to simply as PVA graft) showed tubular structure with apparent even wall thickness (Figure 1A; Table 1) and random roughness along its luminal surface (Figure 1B).

### Blood Compatibility of Submillimeter PVA Vascular Grafts

**Figure 2:**
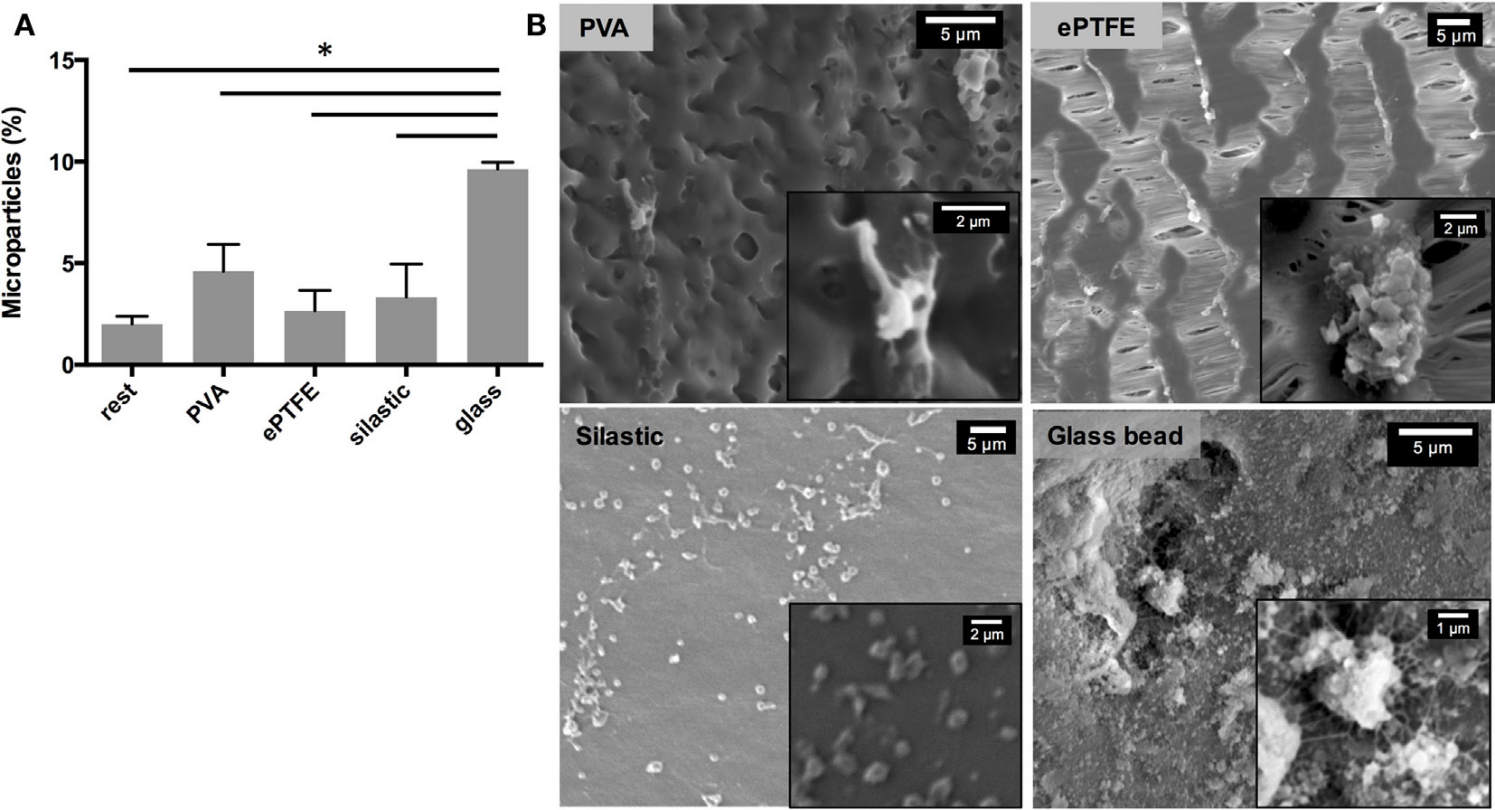
***In vitro* blood compatibility of PVA vascular grafts**. **(A)** Percentage of microparticles expressing GPIIb/IIIa receptor released from total platelet count after contacting various surfaces (*n* = 3). *Denotes statistical significance with two-way ANOVA. **(B)** Morphology of platelets attached to various surfaces observed with SEM. Insets show higher magnification images of platelets.

*In vitro* blood compatibility assay was performed to determine platelet microparticle release and platelet morphology after contact with PVA grafts (Figure 2). Platelet microparticle release, as measured by the expression of the GPIIb/IIIa marker, is an important indicator of platelet activation (Gemmell et al., 1997). Platelets incubated in PVA grafts showed a statistically similar microparticle release profile with that of ePTFE, a material widely used for vascular grafts (Figure 2A). PVA also showed statistically similar platelet microparticle expression with Silastic, an inert material often used for blood-contacting tubing, and the resting control that did not contact any foreign material. Significantly, both PVA and ePTFE showed significantly lower microparticle release compared with the highly thrombogenic collagen-coated glass. PVA and ePTFE showed statistically similar microparticle release with the rest group, indicative of non-activated platelets (Yim et al., 2007). SEM further indicated the thrombogenic potential of collagen-coated glass through the fibrotic and clumped appearance of adhered platelets (Figure 2B). Meanwhile, platelets were sparse on the PVA surface and less fibrous extensions (Figure 2B). Platelets that contacted ePTFE and silastic surfaces were rounder, indicative of the inactive platelet phenotype.

### Implantation of Submillimeter PVA Grafts in Rabbit Femoral Artery

PVA grafts were anastomosed to the left rabbit femoral artery with multilevel arterial occlusion (Figures S3–S5 in Supplementary Material). The multilevel occlusion of the femoral artery was induced to create a challenging environment to test the patency of submillimeter PVA grafts. After implantation of PVA grafts (Table 2), all PVA subjects had normal appearance and mobility 2 days postoperatively. All PVA subjects were alert and responsive with physiological posture and gait at the endpoint. Range of motion of all ankle joints and muscle tone were not affected. No signs of inflammation or infection of the surgical area were seen, indicating the bioinertness of the PVA grafts.

**Table 2:** **Summary of PVA graft implantation**.

Subject	Endpoint (days after surgery)	Diameter of PVA graft used (mm)	Patency at endpoint
PVA1	15	0.9	Patent
PVA2	17	1	Patent
PVA3	14	1	Occluded

**Figure 3:**
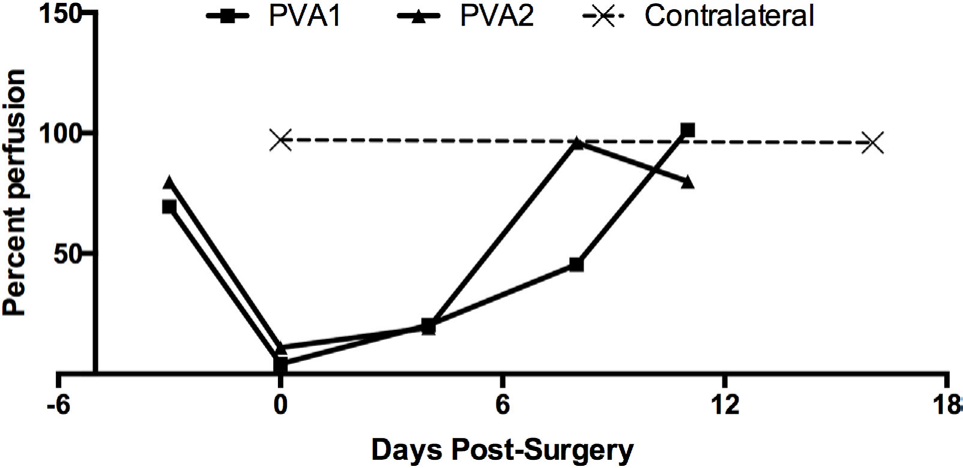
**Recovery of hindlimb surface perfusion after PVA graft implantation**. Percent perfusion on PVA group given as the ratio of surface perfusion between dorsal foot of implanted limb and contralateral dorsal foot. Percent perfusion was obtained at different timepoints after PVA graft implantation (day 0).

The paw surface perfusion, which represents the overall flow contributed by both graft luminal patency and hindlimb collateral formation, was also measured (Figure 3). A notable minimum of percent perfusion was observed in PVA subjects immediately postoperative (day 0; PVA1 4.24% and PVA2 11.0%), indicative of the occlusion induced in the left femoral artery. More importantly, the percent perfusion on patent PVA grafts achieved or exceeded its preoperative level at endpoint (preoperative: PVA1 69 and PVA2 80%; endpoint: PVA1 101.3 and PVA2 80.0%), indicating arterial recovery through graft patency and collateral network formation. In one instance, percent perfusion exceeded the level of the contralateral group.

**Figure 4:**
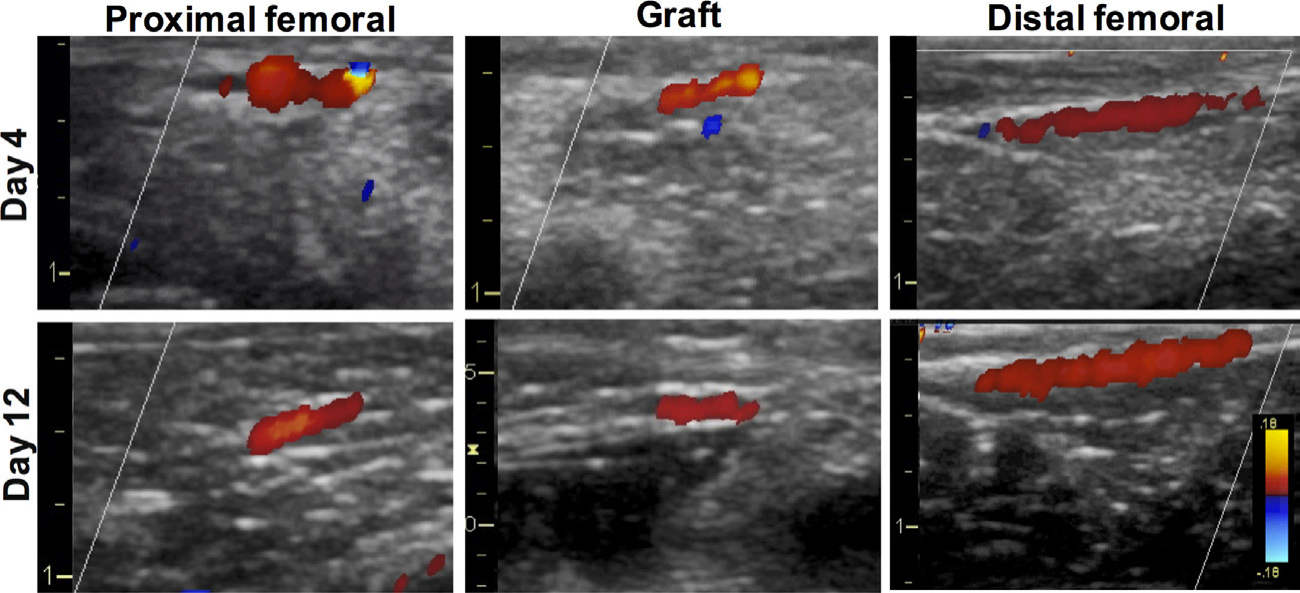
**Estimation of PVA graft patency using ultrasound Doppler**. Representative ultrasound Doppler images were obtained from PVA1 limb. Ultrasound Doppler images were obtained from various areas of the femoral artery, including the graft, at both 4 and 12 days postsurgery. Color scale represents velocity direction, where red indicates blood flow from left to right of the artery.

**Figure 5:**
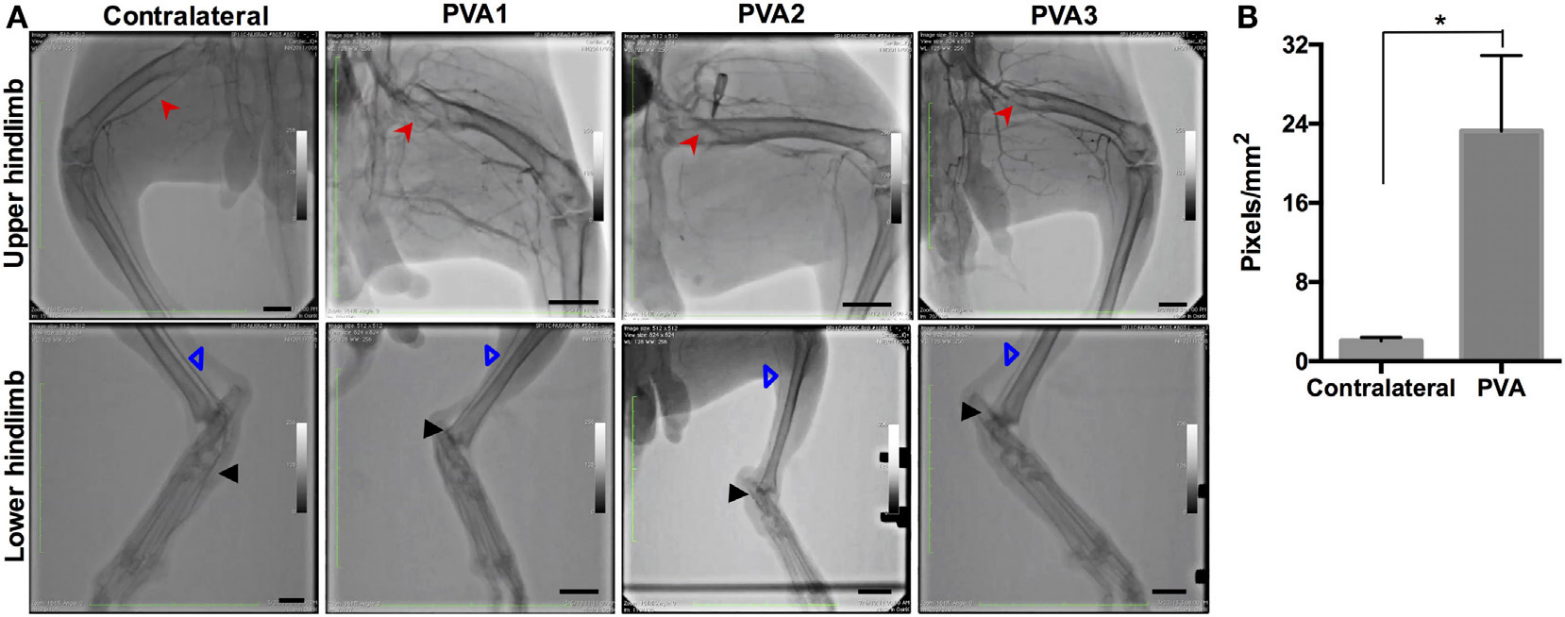
**Patency of femoral, saphenous, and metatarsal arteries, and PVA graft in PVA limbs**. **(A)** Hindlimb perfusion was assessed by angiography. All PVA hindlimbs (*n* = 3) showed extensive collateral network proximally, while blood flow through the saphenous artery was maintained distally. Two out of three PVA grafts remained patent after implantation. A representative angiography from contralateral group (*n* = 5) demonstrated full patency of femoral artery and saphenous artery. Red arrow denotes PVA graft. Blue arrow denotes saphenous artery. Black arrow denotes metatarsal artery. Scale bar = 1 cm. **(B)** Digital examination of angiographs to measure total collateral vessel occupancy in PVA and contralateral group. Collateral vessel occupancy was measured from total pixel area of collaterals normalized to hindlimb area. *Denotes statistical difference using one-way ANOVA.

Ultrasound Doppler was used as a means to estimate graft patency *in situ* (Figure 4). Ultrasound Doppler images showed continuous blood flow everywhere for PVA1 at both 4 and 12 days postoperatively. Normal blood flow was observed throughout the femoral artery and the PVA graft. The PVA graft showed no leakage and no dilatation at both days of observation. Endpoint angiography confirmed the patency of two out of three PVA grafts, giving a primary patency rate of 67% (Figure 5A). Significantly, all PVA limbs showed blood flow through the saphenous artery and metatarsal arteries using angiography. Analysis of angiograms showed that PVA limbs exhibited significantly higher pixel occupancy of blood vessels and more extensive collateral network as opposed to contralateral controls (Figure 5B).

**Figure 6:**
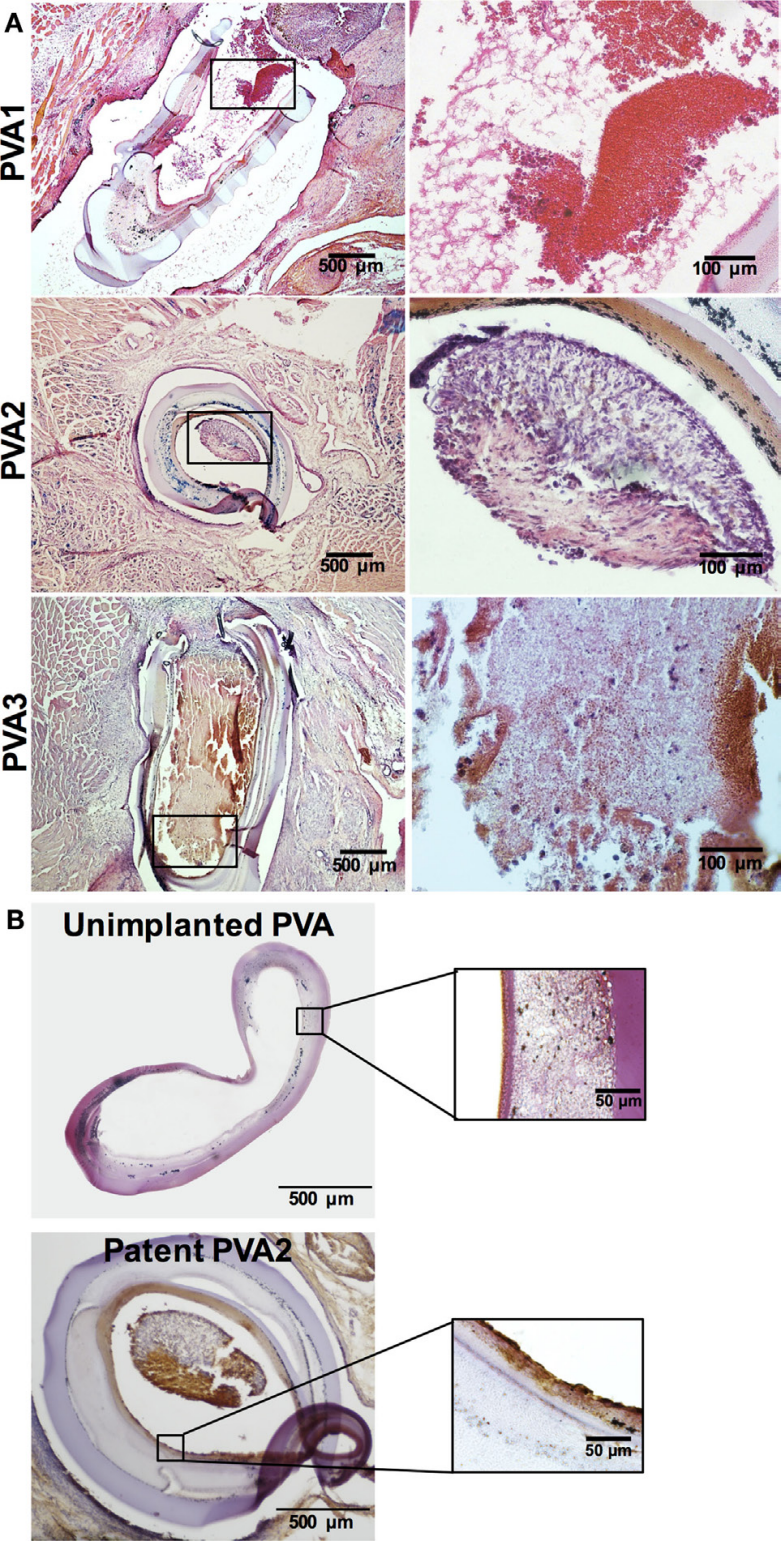
**Histological analysis of PVA vascular grafts**. **(A)** Morphology of explanted PVA vascular grafts (*n* = 3). Hematoxylin and eosin (H&E) staining of sections of both patent (PVA1 and PVA2) and occluded PVA (PVA3) grafts (40×). **(B)** Endothelialization of patent PVA small diameter graft. Positive staining (brown) for CD31 was absent in unimplanted PVA and present in PVA vascular graft after 17 days postimplantation (40×). Insert shows higher magnification (100×).

Explanted PVA grafts showed that PVA1 showed some residual blood cells in the lumen (Figure 6A). In contrast, luminal tissue in PVA2 graft intensely stained with hematoxylin and its contour was similar to that of the graft wall, while the lumen of graft from PVA3 was completely filled with a thrombus (Figure 6A). While an unimplanted PVA graft did not show any positive signal, the entire luminal surface of the patent PVA2 graft indicated the presence of endothelial cells (Figure 6B). Masson’s trichrome staining also showed highly organized collagen fibers deposited in the area surrounding the PVA graft (Figure S6A in Supplementary Material).

**Figure 7:**
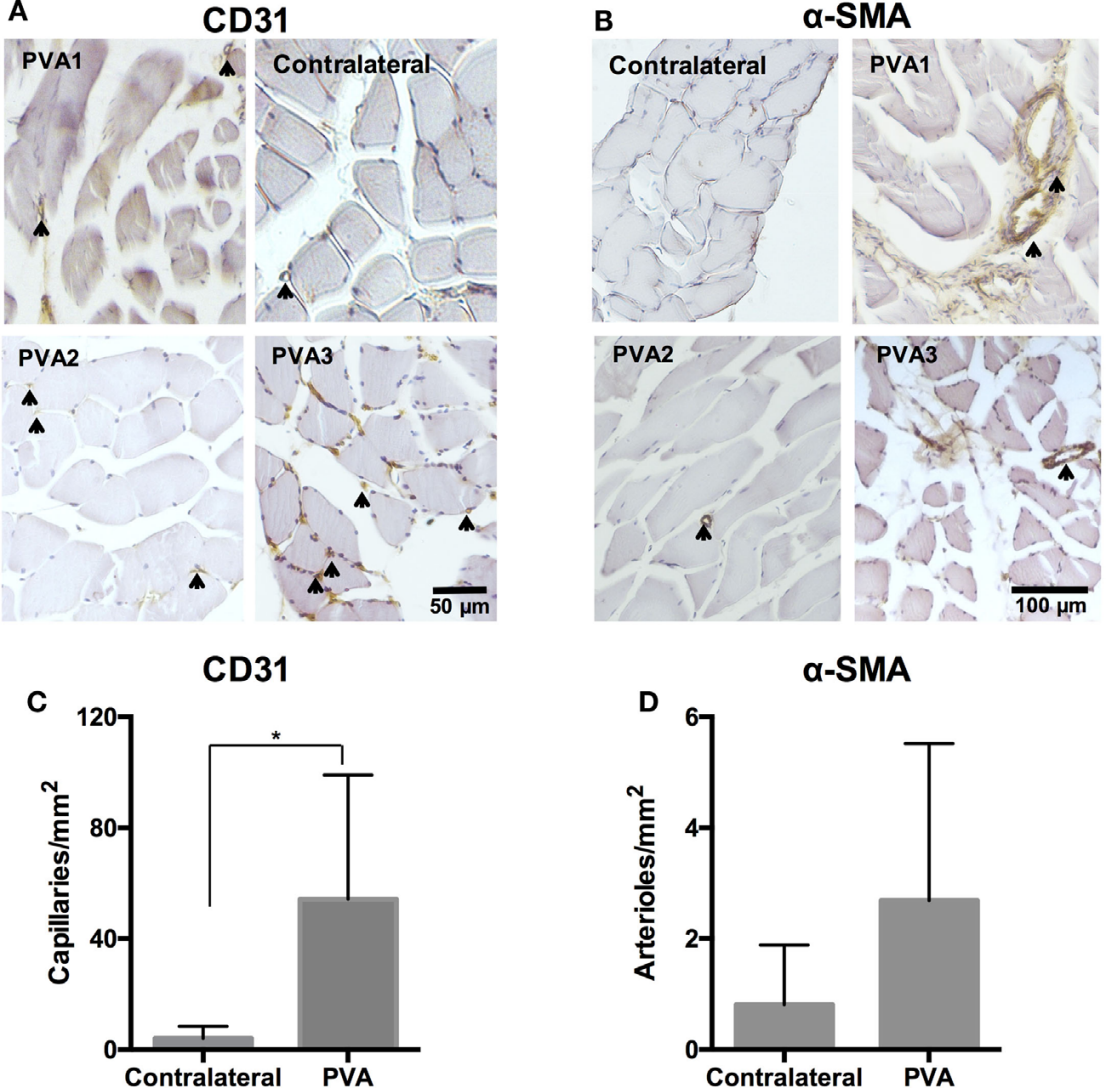
**Capillary and arteriole density in hindlimb of PVA group**. Sections of hindlimb muscle from PVA group (*n* = 3) and contralateral group (*n* = 5) were stained for CD31 [**(A)**, 200×] or α-smooth muscle actin (α-SMA) [**(B)**, 100×] antigen. Vessels positive for antigen are denoted by black arrows and brown staining. **(C,D)** Quantification of vessel density per unit area. *Denotes statistical significance using one-way ANOVA. No statistical differences in arteriole density were observed between groups.

Hindlimb muscles were also assessed for evidence of arterial recovery. Muscle from PVA2 showed signs of recovery from ischemia, denoted by nuclei in the center of muscle fibers (Figure S6B in Supplementary Material). Hindlimb muscles in PVA limbs were bundled and rounded with uniform size (Figures 7A,B). Capillary density was significantly higher in PVA limbs compared to contralateral controls (Figure 7C). Additionally, mature collaterals surrounded by α-SMA-positive cells were increased in PVA, as opposed to the contralateral group (Figure 7D).

## Discussion

Microvascular surgery is becoming more prevalent in the field yet alternatives to the use of autologous submillimeter grafts have not yet fully developed. There is a dearth of clinically efficacious artificial submillimeter diameter vascular grafts. The primary causes of failure of vascular grafts at this size scale are thrombogenicity and material mismatch (particularly, mismatch of diameter and compliance) with the native artery. PVA is commonly used for biomedical applications due to its bioinertness, its ability to imbibe water without compromising mechanical integrity and its tunable mechanical properties. To this end, PVA grafts with submillimeter diameter were mechanically tuned to create a new microvascular graft candidate.

At the microvascular size scale, surgery becomes increasingly difficult and increases risk of blood flow stasis, platelet activation and thrombosis (Sarkar et al., 2006). Nonetheless, the characteristics of PVA grafts were suitable to facilitate anastomosis at this dimension and maintain short-term patency. First, close approximation of the PVA graft mechanical properties, especially the diameter and the compliance, to the rabbit femoral artery likely aided maintenance of normal blood flow and propagation of pulsatile flow. In addition, PVA grafts were easily molded into submillimeter dimensions without loss of mechanical integrity; this contributed to the improved outcome of the anastomosis by minimizing manipulation of the native vessel, which is fragile and susceptible to spasm and thrombosis during clamping (Fujimaki et al., 1977; Siemionow, 1987). Spontaneous luminal opening of PVA graft coupled with its elasticity, suture retention, and overall ease of handling likewise aided the anastomosis, which invariably contributed to graft patency (Siemionow, 1987; Chen et al., 2001). In contrast, the use of the relatively stiff vascular graft made of ePTFE with 1 mm ID showed a dismal 8% patency rate after 2 weeks in the rabbit femoral artery despite a good caliber match (Lidman et al., 1980). Ganske et al. (1982) reported even more drastic outcomes of thrombosis within 48 h postimplantation of ePTFE grafts implanted in the same location. The group further reported that the only patent ePTFE graft showed good tissue attachment but without any indication of endothelialization.

Second, improved resistance to thrombosis and patency can also be attributed to the low thrombogenic potential *in vitro* and endothelialization *in vivo* of the submillimeter PVA vascular grafts. Flat PVA films were previously demonstrated by Ino et al. (2013) to induce similar platelet adherence and thrombin generation with the classic vascular graft material ePTFE. Recently, PVA graft thrombogenicity was tested using a baboon *ex vivo* assay, a more physiologically relevant system that mimics the hydrostatics, hemodynamics and blood chemistry found in small diameter vascular grafts after implantation (Cutiongco et al., 2015a). The authors have reported that PVA grafts with diameter of ~3.75 mm have a lower platelet accumulation profile and total fibrin deposition compared with ePTFE. Thus, tuning the mechanical properties of submillimeter diameter PVA grafts did not deteriorate its low thrombogenic potential.

After 2 weeks of implantation, we observed the patency of 67% of PVA grafts. In at least one of the grafts, an endothelial layer was observed on the luminal surface. The observations are made more remarkable by the fact that the animal subjects did not receive any anticoagulant or antithrombotic regimen. Overall, the mechanical and surface properties of submillimeter diameter PVA graft are strongly advantageous for clinical application in microvascular surgery.

However, PVA vascular grafts are far from universal in its application in microvascular surgery. Natural tapering compounded with variation in blood vessel caliber may cause mismatch at the distal anastomosis. The occlusion of PVA2 graft, which showed possible intimal hyperplastic tissue toward the distal end, supported this notion. It may be necessary to create PVA grafts with gradual tapering to accommodate natural changes in vessel diameter.

In addition to accommodating caliber, changes in compliance, tensile strength, and other mechanical properties should be accommodated. PVA can theoretically be adapted to accommodate multiple types of arteries from different anatomical location. Here, cross-linking degree of PVA was modified by manipulating the cross-linking temperature. PVA cross-linking is speculated to depend on the local concentration of alkaline, which is highly dependent on water evaporation rate. The myriad types of PVA available, with different acetylation degrees and molecular weight, present flexibility in the characteristics of the hydrogel obtained. For instance, this study and that of Ino et al. (2013) cross-linked PVA with different acetylation degrees, resulting in PVA with different material properties. The crosslinker STMP and the alkaline content contributed by sodium hydroxide can also be easily modified to control the rate and extent of cross-linking between the PVA chains and STMP. By increasing STMP and sodium hydroxide content, Chaouat et al. (2008) PVA graft (2 mm ID) matched mechanical properties of the rat abdominal aorta. The flexibility of the material and the cross-linking conditions make PVA an outstanding material that can potentially be used as an off-the-shelf vascular graft for various vascular applications.

It should be noted that Chaouat et al. has previously shown patency of PVA grafts with millimeter dimensions in the rat abdominal aorta. While both rat abdominal aorta and rabbit femoral artery share similar diameters (Byrom et al., 2010) different hemodynamics and hematology of the animal species may contribute to dissimilar graft performance. The high blood flow and shear stress in the aorta, combined with the decreased blood coagulation profile, possibly led to better patency in the rat model. In contrast, rabbits have coagulation profile more closely resembling those of humans (Byrom et al., 2010). Furthermore, the rabbit femoral artery has reduced blood flow from the multilevel arterial occlusion. It is known that increasing severity of distal arterial occlusion is correlated with lower graft patency and limb salvage in humans (O’Mara et al., 1981; Toursarkissian et al., 2002). Though the etiology is not well understood, the main suspect for graft failure in presence of poor blood outflow is the hemodynamic changes caused by distal vessel occlusion. The patency of the submillimeter PVA graft observed in this stringent rabbit model further exemplifies the usefulness of this graft for vascular applications.

## Author Contributions

EY, JH, CLV and JP were responsible for conception and design of the study. MC, MK, JP, MY, JY, AR, and EY performed data collection. MC, MK, CLV, AR, JH, and EY were responsible for data analysis and interpretation. MC and EY were mainly responsible for writing the manuscript. All authors approve this final version of the article.

## Conflict of Interest Statement

The authors declare that the research was conducted in the absence of any commercial or financial relationships that could be construed as a potential conflict of interest.

## References

[B1] Barnes R. W. (1980). Hemodynamics for the vascular surgeon. Arch. Surg. 115, 216–223. 10.1001/archsurg.1980.01380020082021 7356839

[B2] Barzin J. Madaeni S. S. (2007). Hemodialysis membranes prepared from poly(vinyl alcohol): effects of the preparation conditions on the morphology and performance. J. Appl. Polym. Sci. 104, 2490–2497. 10.1002/app.25627

[B3] Byrom M. J. Bannon P. G. White G. H. Ng M. K. C. (2010). Animal models for the assessment of novel vascular conduits. J. Vasc. Surg. 52, 176–195. 10.1016/j.jvs.2009.10.080 20299181

[B4] Chaouat M. Le Visage C. Baille W. E. Escoubet B. Chaubet F. Mateescu M. A. (2008). A novel cross-linked poly(vinyl alcohol) (PVA) for vascular grafts. Adv. Func. Mater. 15, 2855–2861. 10.1002/adfm.200701261

[B5] Chen Y. X. Chen L. E. Seaber A. V. Urbaniak J. R. (2001). Comparison of continuous and interrupted suture techniques in microvascular anastomosis. J. Hand. Surg. 26, 530–539. 10.1053/jhsu.2001.22933 11418920

[B6] Cutiongco M. F. A. Anderson D. E. J. Hinds M. T. Yim E. K. F. (2015a). In vitro and ex vivo hemocompatibility of off-the-shelf modified poly(vinyl alcohol) vascular grafts. Acta Biomater. 25, 97–108. 10.1016/j.actbio.2015.07.039 26225735 PMC4762273

[B7] Cutiongco M. F. A. Choo R. K. T. Shen N. J. X. Chua B. M. X. Sju E. Choo A. W. L. (2015b). Composite scaffold of poly(vinyl alcohol) and interfacial polyelectrolyte complexation fibers for controlled biomolecule delivery. Front. Bioeng. Biotechnol. 3:3 10.3389/fbioe.2015.00003 25692128 PMC4315105

[B8] Cutiongco M. F. A. Goh S.-H. Aid-Launais R. Le Visage C. Yee L. H. Yim E. K. F. (2016). Planar and tubular patterning of micro and nano-topographies on poly(vinyl alcohol) hydrogel for improved endothelial cell responses. Biomaterials 84, 184–195. 10.1016/j.biomaterials.2016.01.036 26828683

[B9] Doi K. Tominaga S. Shibata T. (1977). Bone grafts with microvascular anastomoses of vascular pedicles: an experimental study in dogs. J. Bone Joint Surg. Am. 59, 809–815. 908705

[B10] Fathi E. Nassiri S. M. Atyabi N. Ahmadi S. H. Imani M. Farahzadi R. (2013). Induction of angiogenesis via topical delivery of basic-fibroblast growth factor from polyvinyl alcohol-dextran blend hydrogel in an ovine model of acute myocardial infarction. J. Tissue Eng. Regen. Med. 7, 697–707. 10.1002/term.1460 22674791

[B11] Feng S. Chen H. Liu Y. Huang Z. Sun X. Zhou L. (2013). A novel vitreous substitute of using a foldable capsular vitreous body injected with polyvinylalcohol hydrogel. Sci. Rep 3, 1838. 10.1038/srep01838 23670585 PMC3653144

[B12] Fujimaki A. O’Brien B. Kurata T. Threlfall G. (1977). Experimental micro-anastomosis of 0.4-0.5 mm vessels. Br. J. Plast. Surg. 30, 269–272. 10.1016/0007-1226(77)90115-1 338070

[B13] Ganske J. G. Demuth R. J. Miller S. H. Buck D. C. Dolph J. L. (1982). Comparison of expanded polytetrafluoroethylene microvascular grafts to autogenous vein grafts. Plast. Reconstr. Surg. 70, 193–201. 10.1097/00006534-198208000-00013 7100308

[B14] Gemmell C. H. Yeo E. L. Sefton M. V. (1997). Flow cytometric analysis of material-induced platelet activation in a canine model: elevated microparticle levels and reduced platelet life span. J. Biomed. Mater. Res. 37, 176–181. 10.1002/(SICI)1097-4636(199711)37:2<176:AID-JBM5>3.0.CO;2-O 9358309

[B15] Griffin J. R. Thornton J. F. (2005). Microsurgery: free tissue transfer and replantation. Selected Readings Plast. Surg. 10, 1–41.

[B16] Harris J. R. Seikaly H. (2002). Evaluation of polytetrafluoroethylene micrografts in microvascular surgery. J. Otolaryngol. 31, 89–92. 10.2310/7070.2002.18928 12019749

[B17] Iglarz M. Silvestre J. S. Duriez M. Henrion D. Levy B. I. (2001). Chronic blockade of endothelin receptors improves ischemia-induced angiogenesis in rat hindlimbs through activation of vascular endothelial growth factor-no pathway. Arterioscler. Thromb. Vasc. Biol. 21, 1598–1603. 10.1161/hq1001.097065 11597932

[B18] Ino J. M. Sju E. Ollivier V. Yim E. K. F. Letourneur D. Le Visage C. (2013). Evaluation of hemocompatibility and endothelialization of hybrid poly(vinyl alcohol) (PVA)/gelatin polymer films. J. Biomed. Mater. Res. 101, 1549–1559. 10.1002/jbm.b.32977 23846987

[B19] Judet H. Judet J. Gilbert A. (1981). Vascular microsurgery in orthopaedics. Int. Orthop 5, 1–9. 10.1007/s00586-016-4494-4 7275418

[B20] Kobayashi M. Chang Y.-S. Oka M. (2005). A two year in vivo study of polyvinyl alcohol-hydrogel (PVA-H) artificial meniscus. Biomaterials 26, 3243–3248. 10.1016/j.biomaterials.2004.08.028 15603819

[B21] Lamponi S. Leone G. Consumi M. Greco G. Magnani A. (2012). In vitro biocompatibility of new PVA-based hydrogels as vitreous body substitutes. J. Biomater. Sci. Polym. Ed. 23, 555–575. 10.1163/092050611X554499 21310108

[B22] Lanzetta M. (1995). Clinical use of microvascular PTFE grafts. Microsurgery 16, 412–415. 10.1016/j.jacc.2015.11.066 8531645

[B23] Lidman D. H. Faibisoff B. Daniel R. K. (1980). Expanded polytetrafluoroethylene as a microvascular graft: an experimental study. Microsurgery 1, 447–456. 10.1002/micr.1920010607 7452144

[B24] O’Mara C. S. Flinn W. R. Neiman H. L. Bergan J. J. Yao J. S. (1981). Correlation of foot arterial anatomy with early tibial bypass patency. Surgery 89, 743–752. 7245037

[B25] Sarkar S. Salacinski H. J. GG H. Seifalian A. M. (2006). The mechanical properties of infrainguinal vascular bypass grafts: their role in influencing patency. Eur. J. Vasc. Endovasc. Surg. 31, 627–636. 10.1016/j.ejvs.2006.01.006 16513376

[B26] Sarkar S. Schmitz-Rixen T. Hamilton G. Seifalian A. M. (2007). Achieving the ideal properties for vascular bypass grafts using a tissue engineered approach: a review. Med. Biol. Eng. Comput. 45, 327–336. 10.1007/s11517-007-0176-z 17340153

[B27] Shen T. Mitchell G. Morrison W. O’Brien B. (1988). The use of long synthetic microvascular grafts to vascularise free flaps in rabbits. Br. J. Plast. Surg. 41, 305–312. 10.1016/0007-1226(88)90116-6 3382857

[B28] Siemionow M. (1987). Evaluation of long-term patency rates of different techniques of arterial anastomosis in rabbits. Microsurgery 8, 25–29. 10.1002/micr.1920080108 3295465

[B29] Silvestre J. S. Mallat Z. Duriez M. Tamarat R. Bureau M. F. Scherman D. (2000). Antiangiogenic effect of interleukin-10 in ischemia-induced angiogenesis in mice hindlimb. Circ. Res. 87, 448–452. 10.1161/01.RES.87.6.448 10988235

[B30] So T. Y. (1998). Freeze-dried vessels as interpositional grafts in microsurgery. Microsurgery 18, 248–255. 10.1002/(SICI)1098-2752(1998)18:4<248::AID-MICR7>3.0.CO;2-F 9779637

[B31] Toursarkissian B. D’Ayala M. Stefanidis D. Shireman P. K. Harrison A. Schoolfield J. (2002). Angiographic scoring of vascular occlusive disease in the diabetic foot: relevance to bypass graft patency and limb salvage. J. Vasc. Surg. 35, 494–500. 10.1067/mva.2002.120046 11877697

[B32] Uchida N. Emoto H. Kambic H. Harasaki H. Chen J. F. Hsu S. H. (1989). Compliance effect on patency of small diameter vascular grafts. ASAIO Trans. 35, 556–558. 10.1097/00002480-198907000-00124 2597533

[B33] Wan W. K. Campbell G. Zhang Z. F. Hui A. J. Boughner D. R. (2002). Optimizing the tensile properties of polyvinyl alcohol hydrogel for the construction of a bioprosthetic heart valve stent. J. Biomed. Mater. Res. 63, 854–861. 10.1002/jbm.10333 12418034

[B34] Yim E. K. F. Liao I. C. Leong K. W. (2007). Tissue compatibility of interfacial polyelectrolyte complexation fibrous scaffold: evaluation of blood compatibility and biocompatibility. Tissue Eng. 13, 423–433. 10.1089/ten.2006.0113 17518574 PMC2440513

