# Supplementary material for: Submillimeter Diameter Poly(Vinyl Alcohol) Vascular Graft Patency in Rabbit Model
Source: Front Bioeng Biotechnol. 2016 Jun 8;4:44. doi: 10.3389/fbioe.2016.00044 (PMC4896917; doi:10.3389/fbioe.2016.00044)
Supplement: Supplementary file 1 [file Data_Sheet_1.DOCX]

***Supplementary Material***

**Sub-millimeter diameter poly(vinyl alcohol) vascular graft patency in rabbit model**

**Cutiongco MFA^1,2^, Kukumberg M^1,^^, Peneyra JL^3,^^, Yeo MS^4,5,^^, Yao JY^2^, Rufaihah AJ^6^, Le Visage C^7^, Ho JP^6,8^, Yim EKF^1,2,6,9,^***

^1^Mechanobiology Institute, Singapore, National University of Singapore, Singapore

^2^Department of Biomedical Engineering, National University of Singapore, Singapore

^3^Comparative Medicine, National University of Singapore, Singapore

^4^Division of Plastic, Reconstructive and Aesthetic Surgery, Department of Surgery, National University Health System, Singapore

^5^Plastic, Reconstructive and Aesthetic Surgery Section, Department of General Surgery, Tan Tock Seng Hospital, Singapore

^6^Department of Surgery, Yong Loo Lin School of Medicine, National University of Singapore, Singapore

^7^INSERM, U791, Center for OsteoArticular and Dental Tissue Engineering, Université de Nantes, Nantes, France

^8^Department of Cardiovascular and Thoracic Surgery, National University Health System, Singapore

^9^Department of Chemical Engineering, University of Waterloo, Waterloo, Ontario, Canada

^authors contributed equally

*** Correspondence:** EKF Yim, Department of Chemical Engineering, University of Waterloo, 200 University Avenue West, Waterloo, Ontario, N2L 3G1, Canada

eyim@uwaterloo.ca

1. **Supplementary Data**

## 1.1 Characterization of multi-level occlusion of the rabbit femoral artery

While the field has been rapidly growing to address the critical need for new vascular grafts, the animal models available is still limited to assessing patency, stenosis and endothelialization of vascular grafts as the only parameter to indicate clinical efficacy. The classical hindlimb ischemia model used in vascular graft testing involves surgical ligation of only a major artery (and sometimes its side branches) to induce hindlimb ischemia before revascularization and fails to account for the multi-level nature of vascular occlusion. It is not uncommon for patients receiving vascular grafts to have sub-optimal hemodynamics because of multi-level occlusion (Toursarkissian *et al*., 2002). Herewith, we show a new animal model of persistent ischemia with possibilities for application in screening of new surgical interventions for vascular occlusive disease. A stringent vascular graft model was created to determine the patency of the vascular graft (Figure S1).

First, we assessed the use of the laser Doppler flowmeter for measuring tissue perfusion compared with the standard laser Doppler imager (Figure S2). The laser Doppler flowmeter showed similar trend in tissue perfusion compared with the laser Doppler imager. To create the animal model for vascular graft implantation, the effect of occluding both macro- and micro-circulation on dorsal foot perfusion and femoral artery patency across time was determined. Micro-circulation was occluded using embolic particles, while macro-circulation was disrupted through permanent ligation of left femoral artery. Before surgery, all Ungrafted limbs showed paw surface perfusion within 77-118% (Figure S4; Ungrafted1 76.5%; Ungrafted2 95.8%; Ungrafted3 102%; Ungrafted4 118%). After surgery and induction of the multi-level femoral occlusion, a profound decline in Ungrafted paw surface perfusion to 4-14.7% (Ungrafted1 13.3%; Ungrafted2 4.00%; Ungrafted3 7.91%; Ungrafted4 14.7%) was observed immediately at day 0, after femoral artery ligation and embolization. All Ungrafted limbs then exhibited a general upward trend in perfusion percentage during recovery. However, perfusion percentage did not return to its pre-operative levels even when recovery time was extended, indicating persistence of ischemia. Ungrafted2, Ungrafted3 and Ungrafted4 achieved 82.8%, 55.9% and 62.9% perfusion, respectively, at its endpoint (Figure S3). Notably, Ungrafted1 exhibited high perfusion at day 14 from the applied topical glucocorticoid, which was used as local analgesic and anti-inflammatory to treat self-inflicted wounds (Figure S3B).

Immediately after surgery, all Ungrafted subjects were less prone to ambulation and showed depressed appearance. Low-grade cyanotic skin discoloration of the paw was also observed. At the endpoint, only Ungrafted2 subject had physiological posture and gait. Meanwhile, Ungrafted1 limb exhibited discomfort of the paw one day post-surgery with concurrent low-grade cyanotic skin discoloration. Furthermore, due to the decreased perfusion of the paw and possible ischemic pain from gangrene at 10 days post-operatively, Ungrafted1 subject exhibited self-mutilation behaviour in addition to lacerations caused by decreased proprioception in the paw.

Angiography verified that representative Ungrafted1 and Ungrafted2 limbs had retarded filling beyond the femoral artery and saphenous artery (Figure S4). Quantitative analysis of angiography showed comparable pixel occupancy between Contralateral controls and Ungrafted limbs (Figure S4). On the other hand, the Contralateral control showed blood flow through the femoral, saphenous and metatarsal arteries.

To assess collateral formation, hindlimb muscles were stained against CD31, which denotes endothelial cells on nascent capillaries, and α-SMA, which marks smooth muscle cells on arterioles. Histological analysis of hindlimb muscles surrounding the ligated femoral artery shows muscle fibers with sharp corners and large spatial separation, compared with the round intact muscle bundles of Contralateral controls (Figure S5A and B). Furthermore, there were no statistical differences observed between capillaries and arterioles in Ungrafted limbs compared with the Contralateral controls (Figure S5C and D).

Using the rabbit model with multi-level femoral artery occlusion, we observed extensive collateral network with restoration of distal blood flow in PVA as opposed to Contralateral groups. It is highly plausible that the collateral network provided new blood routes to improve foot perfusion through the saphenous and metatarsal arteries, which are the major vasculature found below the knee in rabbits. Collateral network formation may stem from a wound-healing response initiated by vessel damage during anastomosis (Abaci *et al*., 2010). It is also postulated that mechanical stimulation of the hindlimb in PVA subjects induced an angiogenic response (Gustafsson *et al*., 1999; Shen *et al*., 2009). Mild hypoxia may also stimulate an angiogenic response, as mediated by IL-6 and the inflammatory pathway (Palmer-Kazen *et al*., 2009). It may be worthwhile to determine the cause of increased collateral formation in animals with PVA graft implantation.

1. **Supplementary Figures and Tables**

## Suplementary Tables

| Table S1. Summary of subjects with multi-level femoral artery occlusion (without PVA graft implantation) | |
| --- | --- |
| **Subject** | **Endpoint (days after surgery)** |
| Ligated1 | 15 |
| Ligated2 | 25 |
| Ligated3 | 26 |
| Ligated4 | 29 |

## Suplementary Figures

Figure S1. Induction of multi-level arterial occlusion of the femoral artery and implantation with small diameter (<1mm) poly(vinyl alcohol) (PVA) vascular graft. (A) Overview of rabbit circulatory system. (B) Ligated group (n=4) underwent ligation of femoral artery and embolic particle administration to induce multi-level arterial occlusion (without PVA graft implantation). Red arrow denotes femoral artery administered with embolic particles. (C) Representative image of ligated femoral artery in Ligated limb. Black arrows show ends of ligated femoral artery. Blue arrows denote ends of femoral artery. Green arrow denotes resected femoral artery to be removed. (D) PVA group (n=3) underwent vascular graft anastomosis to left femoral artery with multi-level arterial occlusion. Red arrow denotes femoral artery administered with embolic particles. (E) Representative image of PVA vascular graft after anastomosis in PVA limbs. Proximal and distal are in reference to the position of the PVA vascular graft. The contralateral limbs from both Ligated and PVA groups were used as an internal control (Contralateral, n=7).

Figure S2. Correlation of surface perfusion measurement between laser Doppler flowmetry and imager. Validation of method was performed on two rabbits with PVA vascular graft implantation and treated with aspirin (not included in study). (A, B) shows surface perfusion as measured by the laser Doppler flowmeter and quantified from images. (C, D) shows images obtained from laser Doppler imager. Day 0 denotes administration of embolic particles.

Figure S3. Assessment of the recovery of surface perfusion in dorsal foot of Ligated limbs. (A) Percent perfusion of dorsal foot in Ligated limbs (n=4) at different timepoints after surgery. Percent perfusion is reported as ratio of surface perfusion of dorsal foot of Ligated group to Contralateral dorsal foot. (B) Photomicrograph of gangrenous dorsal foot of Ligated1. Blue dotted line denotes the 100% perfusion level, indicating recovery of hindlimb perfusion. Percent perfusion of Contralateral limb from Figure 3 included for comparison.

Figure S4. Patency of femoral artery and saphenous artery in Ligated limbs assessed by angiography. (A) Femoral artery patency and perfusion of the distal hindlimb of Ligated group (n=2) were assessed by angiography. Red arrow
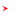
 denotes femoral artery and area of ligation for Ligated limbs. Blue arrow
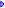
 denotes saphenous artery. Black arrow
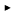
 denotes metatarsal artery. Scale bar = 1 cm. (B) Digital examination of angiographs to measure total collateral vessel occupancy in Ligated group. Collateral vessel occupancy was measured from total pixel area of collaterals normalized to hindlimb area. Pixel occupancy of Contralateral limb from Figure 5 included for comparison. No statistical difference was found between Ligated and Contralateral groups, or between Ligated and PVA groups.

Figure S5. Capillary and arteriole density in Ligated limbs. Sections of hindlimb muscles from Ligated (n=2) limbs were stained for CD31 (A, 200X) or α-SMA (B, 100X) antigen. Vessels positive for antigen denoted by black arrows (A, B) and brown staining. (C, D) Quantified vessel density per unit area. Capillary and arteriole density of Contralateral group from Figure 7 included for comparison. No statistical differences between all samples. No statistical difference in capillary or arterial density was found between Contralateral and Ligated groups, or between PVA and Ligated groups.

Figure S6. Assessment of tissue in the vicinity of patent PVA vascular graft. (A) Masson’s trichrome stain of collagen (blue) surrounding graft. Box denotes area viewed under high magnification (inset, 100X). * denotes graft implantation area. (B) H&E stain of muscle surrounding graft. Black arrows shows muscle fiber with central nucleus (100X).

1. **References^[[1]](#footnote-1)^**

Abaci, H. E., Hanjaya-Putra, D., and Gerecht, S. (2010). “Hypoxia and Matrix Manipulation for Vascular Engineering,” in *Biophysical Regulation of Vascular Differentiation and Assembly Biological and Medical Physics, Biomedical Engineering,* ed: S Gerecht (New York, NY: Springer New York), 127–165. doi:10.1007/978-1-4419-7835-6_7.

Gustafsson, T., Puntschart, A., Kaijser, L., Jansson, E., and Sundberg, C. J. (1999). Exercise-induced expression of angiogenesis-related transcription and growth factors in human skeletal muscle. Am. J. Physiol. 276, H679–85.

Palmer-Kazen, U., Religa, P., and Wahlberg, E. (2009). Exercise in Patients with Intermittent Claudication Elicits Signs of Inflammation and Angiogenesis. Eur. J. Vasc. Endovasc. Surg. 38, 689–696. doi:10.1016/j.ejvs.2009.08.005.

Shen, M., Gao, J., Li, J., and Su, J. (2009). Effect of ischaemic exercise training of a normal limb on angiogenesis of a pathological ischaemic limb in rabbits. Clin. Sci. 117, 201–208. doi:10.1016/S0047-6374(00)00178-0.

Toursarkissian, B., D'Ayala, M., Stefanidis, D., Shireman, P. K., Harrison, A., Schoolfield, J., et al. (2002). Angiographic scoring of vascular occlusive disease in the diabetic foot: relevance to bypass graft patency and limb salvage. *J. Vasc. Surg.* 35, 494–500. doi:10.1067/mva.2002.120046.

1. Provide the doi when available, and ALL complete author names.

   [↑](#footnote-ref-1)
