# Supplementary figures and images for: Submillimeter Diameter Poly(Vinyl Alcohol) Vascular Graft Patency in Rabbit Model
Source: Front Bioeng Biotechnol. 2016 Jun 8;4:44. doi: 10.3389/fbioe.2016.00044 (PMC4896917; doi:10.3389/fbioe.2016.00044)

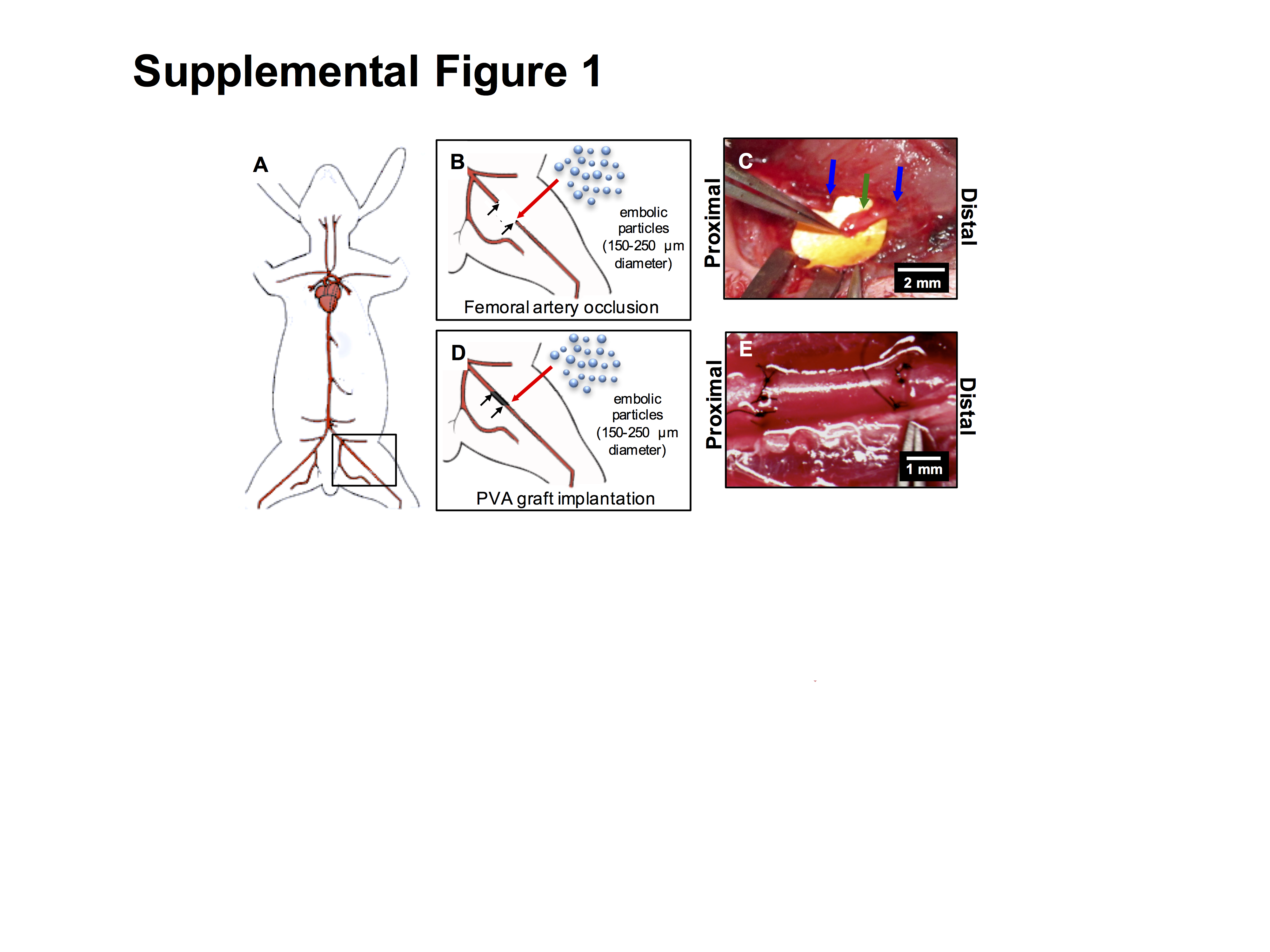

Supplement: Supplementary file 2 [file Image_1.TIFF]

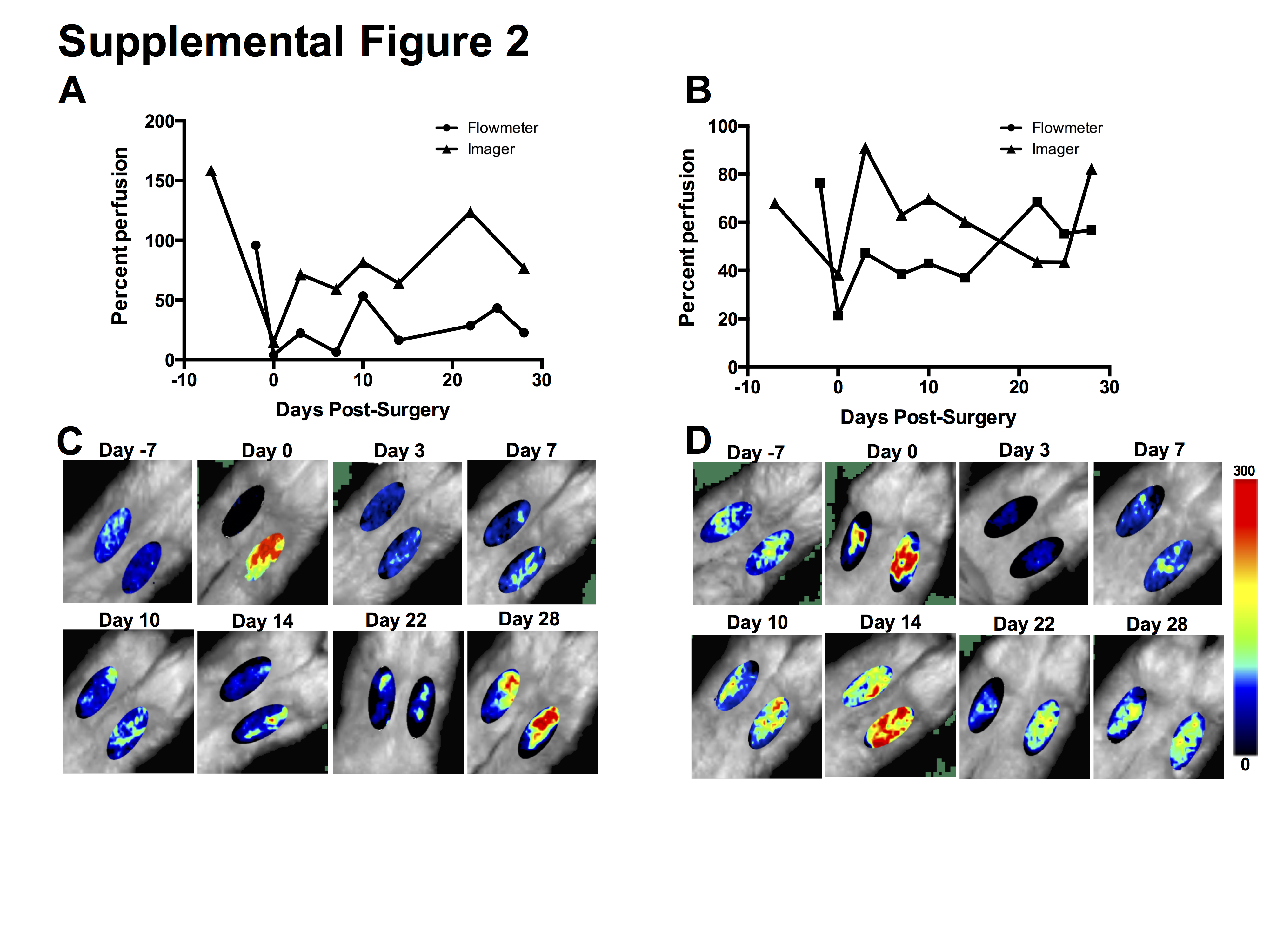

Supplement: Supplementary file 3 [file Image_2.TIFF]

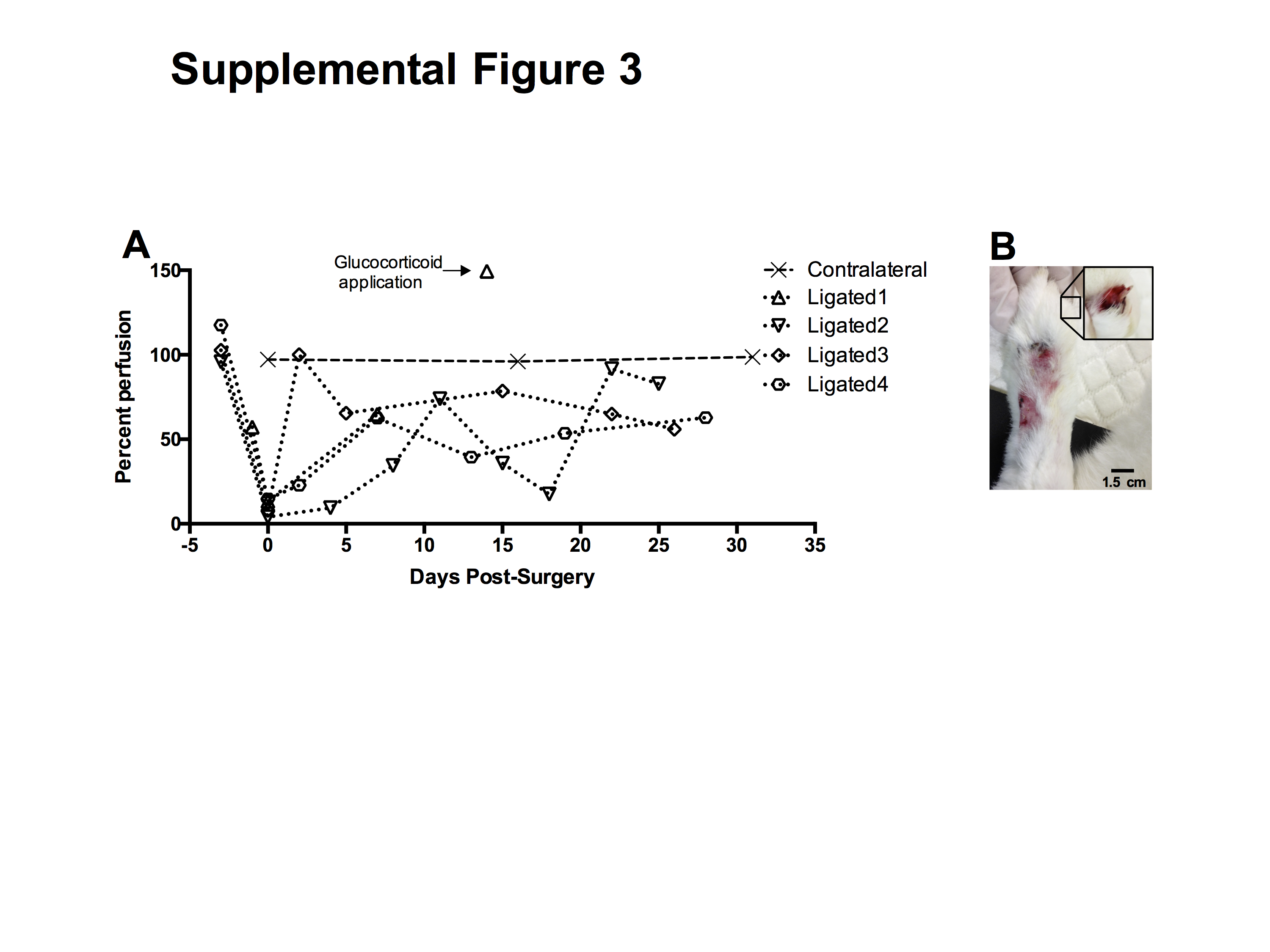

Supplement: Supplementary file 4 [file Image_3.TIFF]

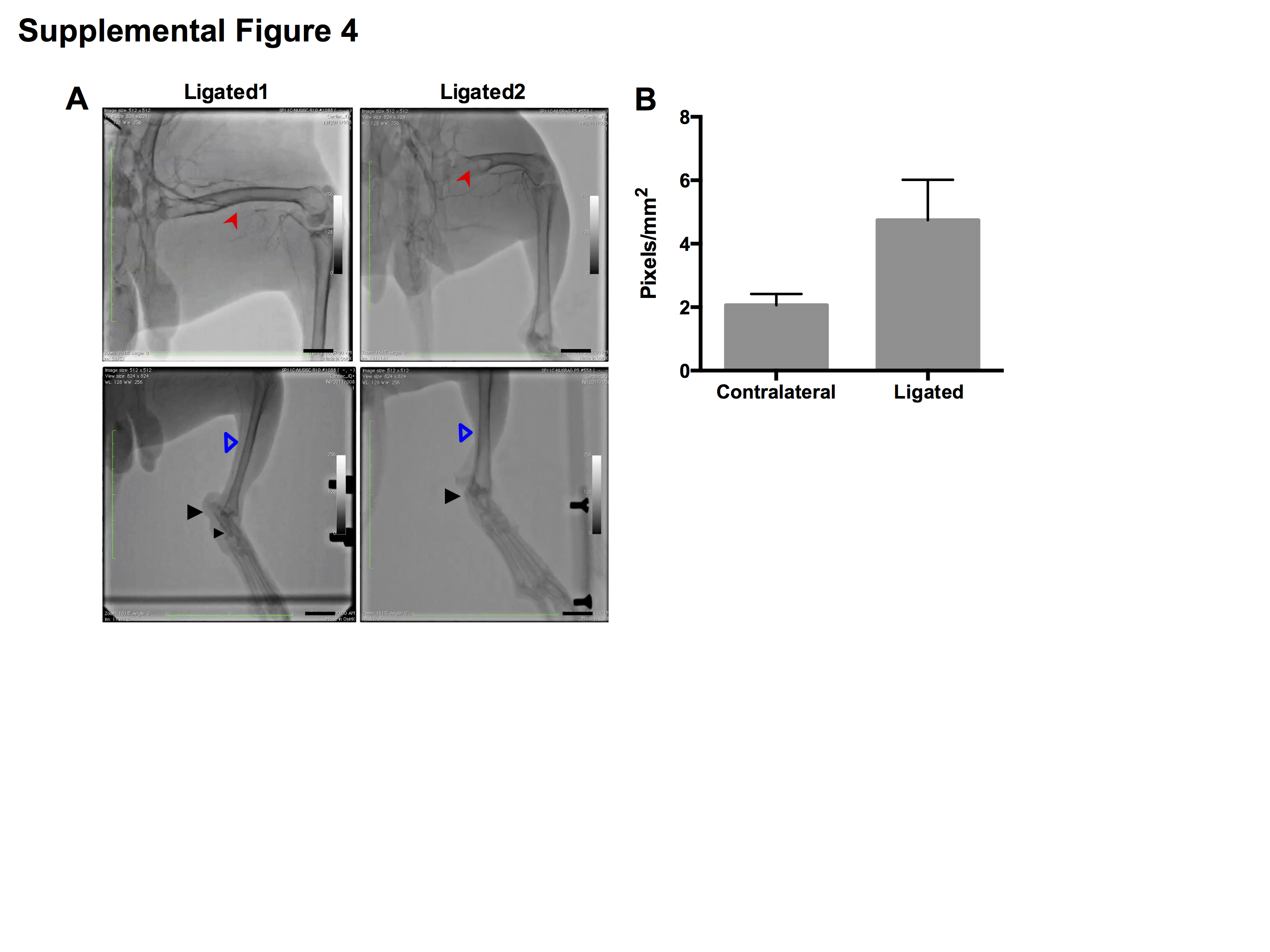

Supplement: Supplementary file 5 [file Image_4.TIFF]

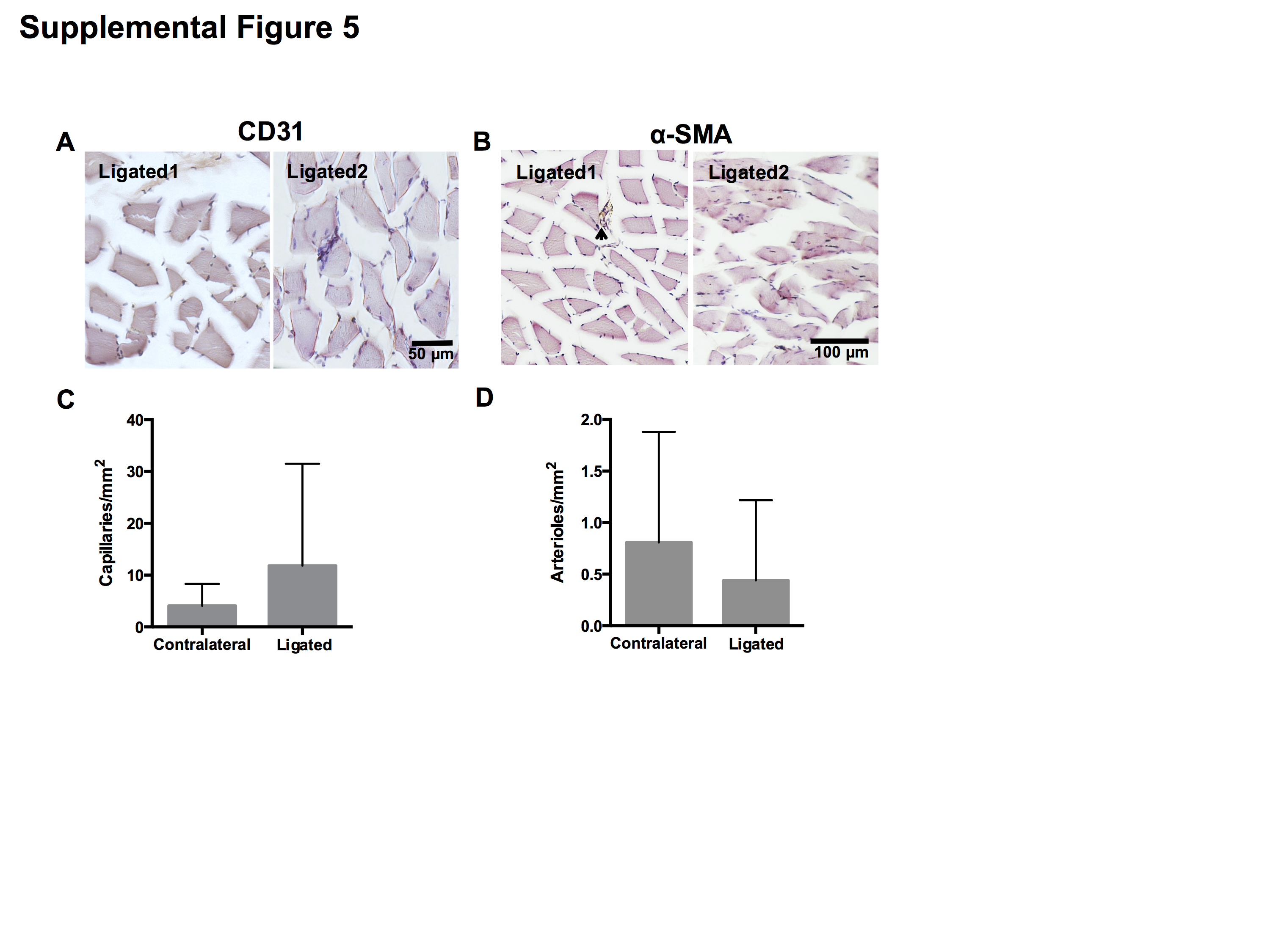

Supplement: Supplementary file 6 [file Image_5.TIFF]

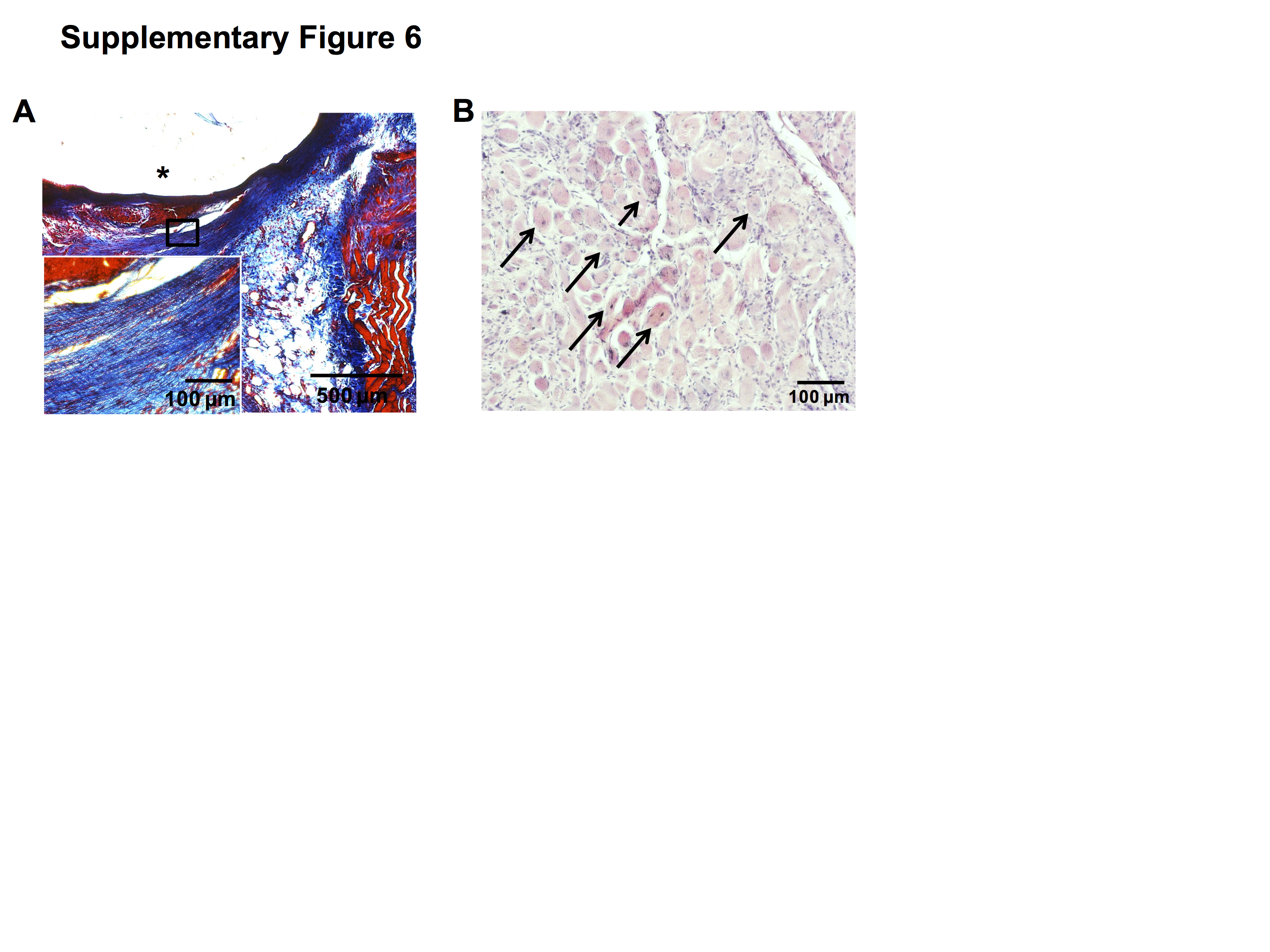

Supplement: Supplementary file 7 [file Image_6.TIFF]
